# ASCA-related antibodies in the blood sera of healthy donors and patients with colorectal cancer: characterization with oligosaccharides related to *Saccharomyces cerevisiae* mannan

**DOI:** 10.3389/fmolb.2023.1296828

**Published:** 2023-12-07

**Authors:** Vadim B. Krylov, Anton N. Kuznetsov, Alina V. Polyanskaya, Pavel V. Tsarapaev, Dmitry V. Yashunsky, Nikolay E. Kushlinskii, Nikolay E. Nifantiev

**Affiliations:** ^1^ Laboratory of Synthetic Glycovaccines, N. D. Zelinsky Institute of Organic Chemistry, Russian Academy of Sciences, Moscow, Russia; ^2^ Laboratory of Glycoconjugate Chemistry, N. D. Zelinsky Institute of Organic Chemistry, Russian Academy of Sciences, Moscow, Russia; ^3^ N. N. Blokhin National Medical Research Center of Oncology, Ministry of Health of the Russian Federation, Moscow, Russia

**Keywords:** mannan, *Saccharomyces cerevisiae*, *Candida albicans*, fungi, antibodies, IgG, human sera, colorectal cancer

## Abstract

Mannans are polysaccharide antigens expressed on the cell wall of different fungal species including *Saccharomyces cerevisiae* and *Candida* spp. These fungi are components of the normal intestinal microflora, and the presence of antibodies to fungal antigens is known to reflect the features of the patient’s immune system. Thus, titers of IgG and IgA antibodies against *Saccharomyces cerevisiae* mannan (ASCA) are markers for clinical diagnostics of inflammatory bowel diseases. The complex organization and heterogeneity of cell-wall mannans may reduce the quality and reproducibility of ELISA results due to interference by different antigenic epitopes. In this research, we analyzed the levels of IgG antibodies in the sera of healthy donors and patients with colorectal cancer using an array of synthetic oligosaccharides related to distinct fragments of fungal mannan. This study aimed to establish the influence of oligosaccharide structure on their antigenicity. Variations in the structure of the previously established ASCA epitope (changing type of linkage, chain length, and the presence of branches) significantly modified the ability of ligands to bind to circulating antibodies in blood sera. The study showed that surface presentation density of the ligand critically affects the results of enzyme immunoassay. The transition from natural coating antigens to their corresponding synthetic mimetics with a defined structure opens new opportunities for improving existing ELISA test systems, as well as developing diagnostic kits with new properties.

## 1 Introduction

**FIGURE 1:**
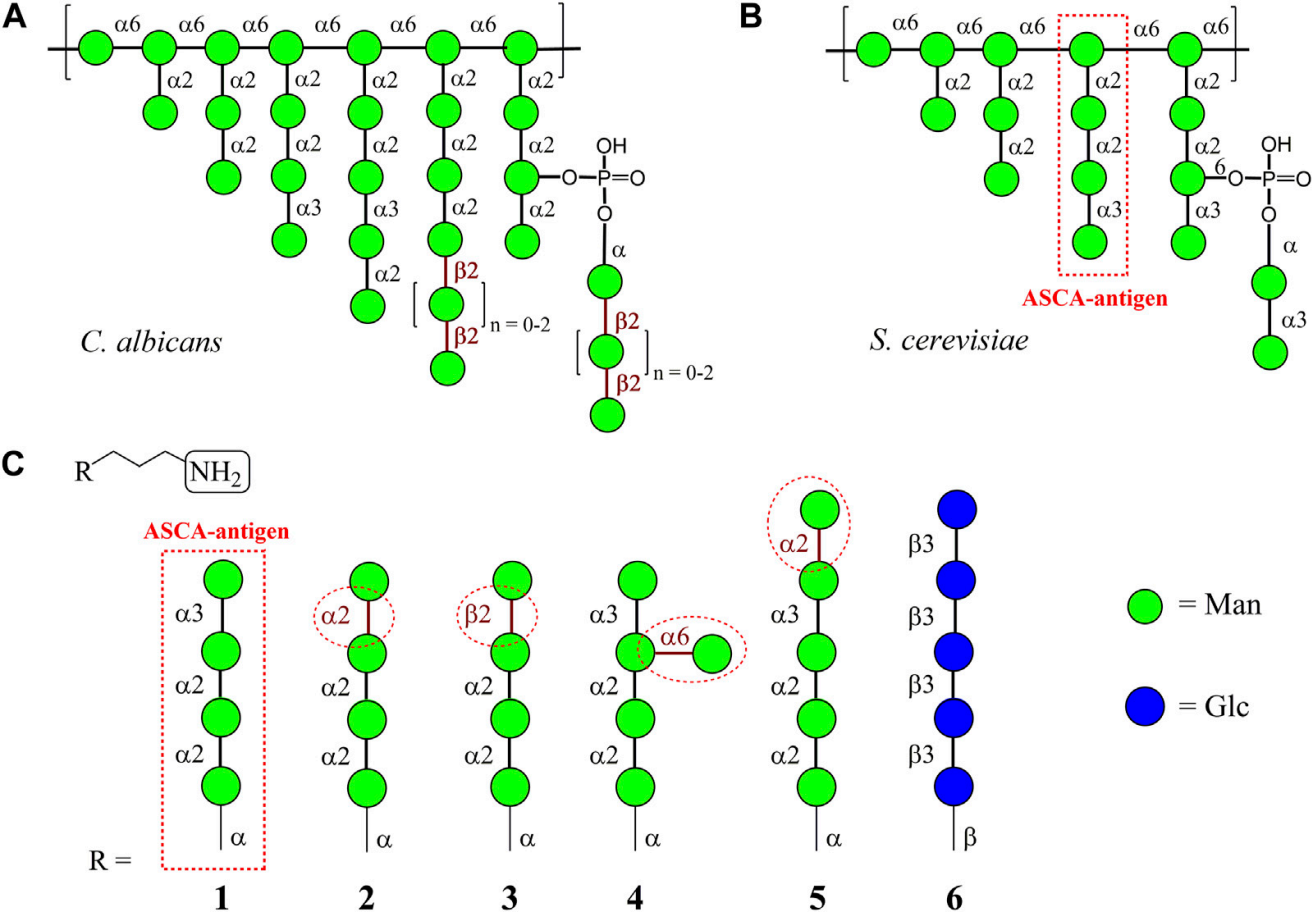
Tentative structures of *C. albicans* (Suzuki, 1997) **(A)** and *S. cerevisiae* (Vinogradov et al., 1998) **(B)** mannans; **(C)** carbohydrate sequences in the oligomannosides R-(CH_2_)_3_NH_2_ **1**–**5** used for glycoarray creation in this work. Penta-β-(1→3)-Glucoside **6**, the fragment of β-D-glucan of the fungal cell wall, was used as the control sample.

Fungal cell-wall polysaccharides are dominant surface antigens stimulating immune reactions in humans (Erwig and Gow, 2016). The heterogeneity and high variability of substructures in polysaccharides lead to a multiplicity of so-called “antigenic factors” (Suzuki, 1997) and the generation of a repertoire of antibodies with varying specificity (Solovev et al., 2023). Moreover, the ability of different carbohydrate structures to elicit an antibody response varies depending on their structure. Thus, the use of oligosaccharide ligands as model antigens with distinct structures related to the fragments of polysaccharide components of the fungal cell wall opens the possibility of a comparative study of their immunological properties (Krylov and Nifantiev, 2020). This was demonstrated by several examples. Particularly, our previous works showed that β-oligomannoside fragments of *Candida albicans* mannan (Figure 1A) generated a higher antibody response than antigenic factors with solely α-linked chains (Solovev et al., 2023). The specificity of the monoclonal antibody EBCA-1 used in sandwich immune assay to detect *Candida* mannan was recently reinvestigated, and it was shown that EBCA-1 recognizes the trisaccharide β-Man-(1→2)-α-Man-(1→2)-α-Man and not homo-α-(1→2)-linked pentamannoside, as reported previously (Jacquinot et al., 1998; Krylov et al., 2021). The study of the immunogenic properties of *Aspergillus fumigatus* galactomannan using a library of synthetic oligosaccharide antigens showed that oligogalactofuranoside fragments but not oligomannoside chains are responsible for immune reaction and elicitation of anti-galactomannan antibodies (Wong et al., 2020).

*Saccharomyces cerevisiae* is the common yeast utilized in biotechnology in many food fermentations and other industrial processes (Parapouli et al., 2020). This yeast is an important part of the normal fungal microbiota and is generally regarded as safe; however, recent studies have provided evidence of its involvement in a range of superficial and systemic diseases (Enache-Angoulvant and Hennequin, 2005).

The first cellular component interacting with the host immune system is the yeast cell wall (Erwig and Gow, 2016). Orlean (2012) describedthe architecture and biosynthesis of the *S. cerevisiae* cell wall in detail. Its main carbohydrate component is a branched mannan (Figure 1B), which amounts to approximately half of the total mass of the cell wall and plays an important role in immune response and its regulation (Orlean, 2012).

The detection of antibodies against *S. cerevisiae* mannan, known as ASCA, is used for the differential diagnosis of inflammatory bowel diseases (Szilagyi et al., 2014) such as Crohn’s disease and ulcerative colitis (Main et al., 1988). A comparative study of different ASCA-detecting assays revealed their moderate sensitivity (41%–76%) and good specificity (86%–98%) (Vermeire et al., 2001). The role of antibodies to *S. cerevisiae* mannan in inflammatory bowel diseases is not clearly determined. However, a growing number of studies have detected high levels of ASCA in patients affected with autoimmune diseases, including antiphospholipid syndrome, systemic lupus erythematosus, type 1 diabetes mellitus, rheumatoid arthritis, spondyloarthritis, and hidradenitis suppurativa (Rinaldi et al., 2013; Maillet et al., 2016; Assan et al., 2020). Probably, an imbalance of immune tolerance to commensal microbiota (e.g., *S. cerevisiae*) can trigger systemic autoimmune disorders (Temme et al., 2021). Thus, anti-*S. cerevisiae* mannan antibodies have high potential as clinical diagnostic markers and require a more detailed study of their immunological properties.

In this paper, we describe the results of screening ASCA-related antibodies in the sera of patients with colorectal cancer and healthy controls using glycoconjugates containing distinct oligosaccharide ligands present in the chains of *S. cerevisiae* mannan. To the best of our knowledge, such comparative studies have never been performed. The used glycoconjugates were selected instead of natural polysaccharides because of substantial variations in ASCA titers between assays from different manufacturers (Vermeire et al., 2001) in studies where polysaccharide antigens were employed.

## 2 Materials and methods

### 2.1 Conjugates of synthetic oligosaccharides with biotin and polyacrylamide

Biotinylated conjugates of ligand 1 (Figure 1C) were chemically synthesized by coupling of parent aminospacered oligosaccharides (Karelin et al., 2007) with an activated biotin derivative containing a hydrophilic hexaethylene glycol linker (Tsvetkov et al., 2011), as previously described (Krylov et al., 2018).

For preparation of polyacrylamide (PAA) conjugates, solutions of parent oligosaccharides (Karelin et al., 2007; Argunov et al., 2011; Karelin et al., 2016; Yashunsky et al., 2016; Krylov and Nifantiev, 2020) (1 eq) and p-nitrophenyl polyacrylate (190 mmol of p-nitrophenol groups per 1 mg; 5, 10, 20, or 40 eq) in dry DMF were incubated for 2 h at 25°C. Subsequently, n-butylamine (2.5 eq) was added, and the mixture was incubated for another 2 h at 25°C. Ethanolamine (20 µL) was added, and the reaction mixture was incubated overnight at room temperature. The reaction mixture was concentrated under reduced pressure on a rotovap, and the dry solid was purified with size-exclusive chromatography on Sephadex LH-20 in MeCN:H_2_O (1:1). PAA conjugate fractions were concentrated under reduced pressure on a rotovap and then lyophilized to yield beige solid. ^1^H NMR spectra of obtained conjugates were recorded in D_2_O on a Bruker AV600 (600 MHz) spectrometer.

### 2.2 Human donor sera

The collection of biological material was carried out in the Laboratory of Clinical Biochemistry at the N. N. Blokhin National Medical Research Center of Oncology of the Ministry of Health of Russia. Prior to use, blood serum samples were stored at −152°C. The study included 30 patients with colorectal cancer (15 women aged 56.5 ± 13.1 and 15 men aged 58.6 ± 8.9 years). In all patients, colorectal cancer was detected for the first time and confirmed by histological examination of the tumor. Straight intestine cancer was diagnosed in five patients, blind intestine cancer in four patients, sigmoid colon cancer in 11 patients, ascending part of the colon in five patients, and descending part of the colon in five patients. Three patients with CRC had stage 1 of the disease, nine had stage II, 12 had stage III, and six had stage IV. According to the histological structure, all tumors are characterized as adenocarcinoma. The control group consisted of 18 healthy donors. The information about patients and healthy donors is summarized in Supplementary Tables S1, S2. The healthy donors included nine women aged 50.1 ± 17.7 years and nine men aged 43 ± 17.1 years. This study was approved by the Ethics Committee of the N. N. Blokhin National Medical Research Center of Oncology of the Ministry of Health of the Russian Federation on 11 May 2022 (Protocol No. 5 as of 05/11/2022). Before screening, human blood sera was diluted 400 times in PBS containing 0.05% Tween-20 and 0.1% BSA.

### 2.3 Glycoarray

Polyacrylamide conjugates of oligosaccharides 1–6 were absorbed on the wells of polystyrene plates (Xema, Russia) (100 µL of a 5 μg/mL solution in PBS) for 24 h at 4°С. Subsequently, the wells were washed with PBS one time and blocked with casein (Sigma-Aldrich, Germany) for 1 h at 37°С. After shaking out and drying, the plates were ready to use. The wells of 96-well avidin-coated plates (Xema, Russia) were coated with biotin-tagged oligosaccharide 1 (Figure 1) (100 µL of 200 pmol/mL solution in PBS containing 0.05% Tween-20 and 0.1% BSA) and then incubated for 2 h at 37°C. After washing three times, the plates were ready to use. The ready-to-use plate provided in the Anti-*Saccharomyces cerevisiae* Kit EV2841-9601G (EUROIMMUN AG, Germany) was used as a natural antigen reference.

The plates were incubated with calibration sera provided in the Anti-*Saccharomyces cerevisiae* Kit EV2841-9601G (EUROIMMUN AG, Germany) or diluted human sera for 45 min at 37°C. After washing three times, the wells were treated with conjugates of anti-human IgG Ab with peroxidase (d5000; IMTEK, Russia) and incubated for 30 min at 37°C. The plates were washed five times, and color was developed using 100 µL of TMB monocomponent substrate (Xema, Russia) for 15 min. The reaction was stopped with 50 µL of 1 M sulfuric acid. Absorbance was measured at 450 nm using a Multiskan GO plate reader (Thermo Fisher Scientific, United States). All measurements were repeated independently and performed twice in triplicate.

### 2.4 Statistical analysis

One-way analysis of variance (ANOVA) was used to compare the differences between six carbohydrate ligands. The antibody levels between the two groups were compared using the Mann–Whitney test. The bars represent the mean values with standard deviation (SD).

## 3 Results

Mannans of *S. cerevisiae* (Vinogradov et al., 1998), *C. albicans* (Suzuki, 1997), and other fungi are highly branched polysaccharide antigens that have common and specific structural fragments (Figures 1A, B). For example, both polysaccharides include fragments related to a similar group of antigenic factors composed of α-(1→2)/α-(1→3)-linked mannoside residues. On the other hand, side chains with β-(1→2)-linked mannose residues, which have the highest impact on antibody response (Sendid et al., 2021), were found only in *Candida* spp. (Singh et al., 2023). The oligomannoside sequence within *S. cerevisiae* mannan corresponding to antibodies associated with Crohn’s disease was assigned to be the following mannotetraoside: Man(1→3)Man(1→2)Man(1→2)Man (Sendid et al., 1996; Chevalier et al., 2005), which is illustrated in Figure 1B. Therefore, the corresponding oligosaccharide **1** (Karelin et al., 2007) was selected in this study as a basis for the creation of structurally related glycoarray (Figure 1C). Ligands **2** (Karelin et al., 2007) and **3** (Karelin et al., 2016) stem from **1** after formally replacing the terminal α-(1→3)-mannoside fragment with α-(1→2)- and β-(1→2)-mannoside units, respectively. Additional glycosylation of ligand **1** leads to the formation of ligands **4** and **5**. Branched oligosaccharide **4** (Argunov et al., 2011) contains the branch at O(6) of the central mannose residue, and pentasaccharide **5** is glycosylated with α-(1→2)-mannose at the terminal end of the original antigen **1**. The β-(1→3)-glucan fragment **6** (Yashunsky et al., 2016) was taken as a representative of another class of fungal cell-wall polysaccharides and used as a negative control ligand.

**FIGURE 2:**
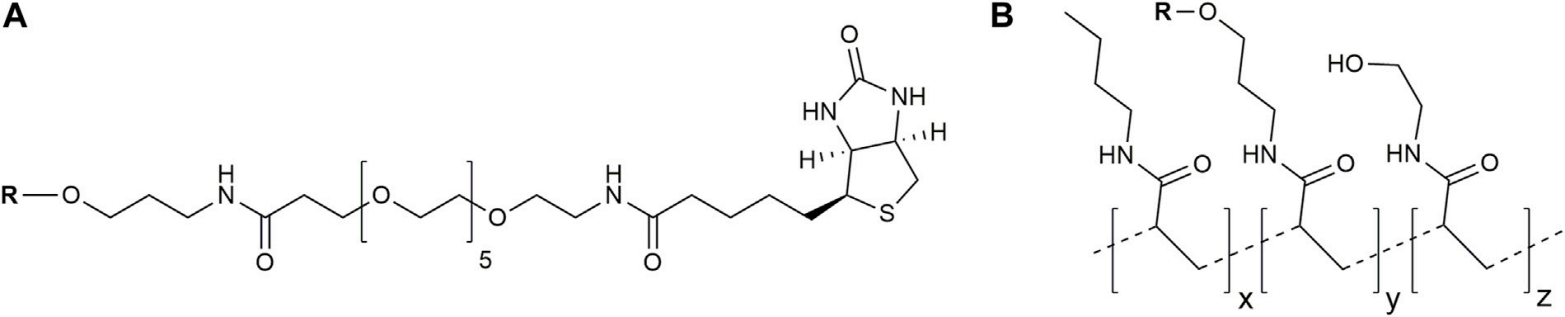
Structures of used glycoconjugates: biotinylated conjugates **(A)** and PAA-based conjugates **(B)** (the tentative structure is shown). R, oligosaccharide ligand.

**FIGURE 3:**
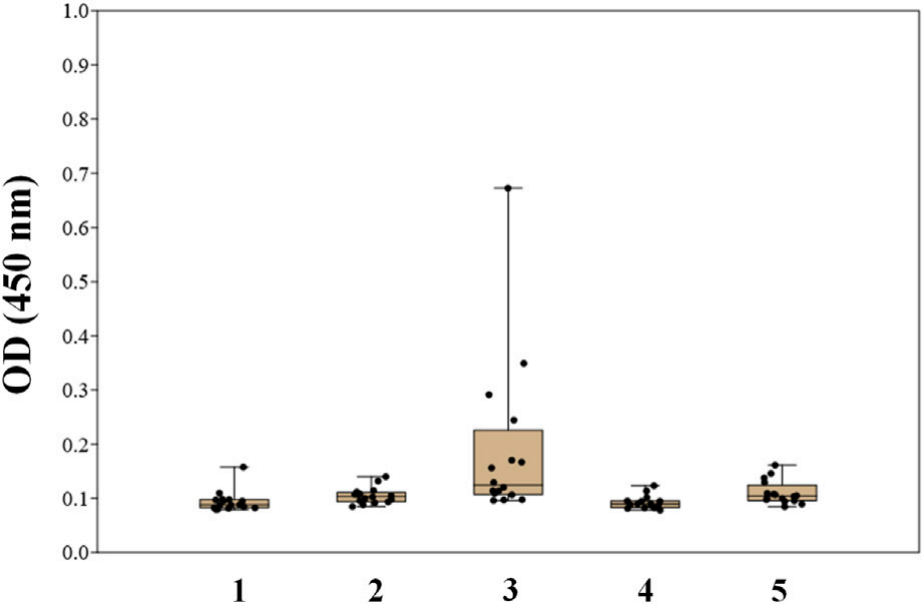
Screening results of IgG antibodies to oligosaccharides **1–5** in the blood sera of healthy donors using biotinylated conjugates of oligosaccharides **1–5** (Solovev et al., 2023).

Two types of glycoconjugates as coating antigens for ELISA studies were used in this work. They included biotinylated conjugates (Figure 2A), which were prepared by coupling of compounds **1**–**6** with activated pentafluorophenyl ester of hexaethylene glycol-tagged biotin (Tsvetkov et al., 2011). Obtained biotinylated oligosaccharides were immobilized on avidin-coated plates and used for sera screening. It appeared that biotinylated α-linked mannosides **1**, **2**, **4**, and **5** did not show any significant signals for healthy donor sera (Figure 3) or for patients with colorectal cancer (Solovev et al., 2023). We hypothesized that loading of oligosaccharide ligands onto microtiter well surfaces with higher density may improve the sensitivity of ELISA and permit better detection of low-affinity antibodies.

**FIGURE 4:**
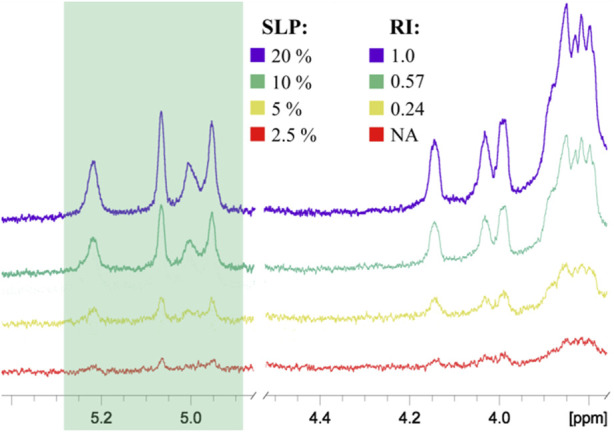
Comparison of ^1^H NMR spectra of PAA conjugates with different percentages of oligosaccharides: SLP, stoichiometric ligand percentage; RI, relative integral intensity of the anomeric signals shown in the green box. All spectra were normalized by the intensity of the PAA matrix signals. The RI of the conjugate with the highest ligand content was taken as 1.00. NA, integration data are not available due to low signal intensity.

To prove this concept, polyacrylamide-based conjugates (see tentative structure in Figure 2B) with different percentages of oligomannoside **1** related to Crohn’s disease marker were obtained (see Section 2.1). The desired content of loaded carbohydrate ligands in synthesized PAA conjugates of 20, 10, 5, and 2.5 mol% was confirmed using ^1^H NMR spectroscopy. Intensities of carbohydrate signals (Figure 4) decreased with a reduction in carbohydrate incorporation, while polymeric matrix signals had a constant profile. Integration of the anomeric signals in ^1^H NMR spectra of conjugates confirmed the content of attached oligosaccharide ligands.

**FIGURE 5:**
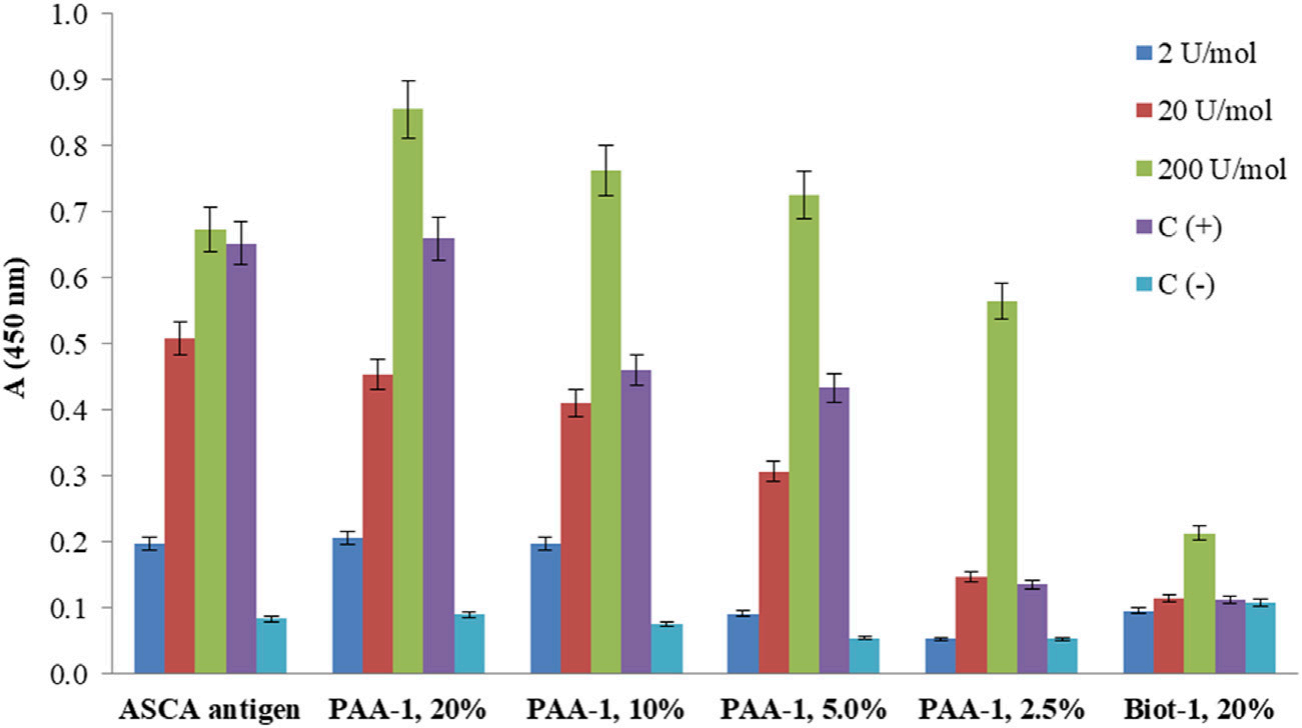
ELISA of calibrant samples (ASCA: 2, 20, and 200 U/mol), ASCA-positive, and ASCA-negative control sera [C(+) and C(−)] using a commercially available diagnostic kit coated with natural ASCA antigen and synthetically prepared coating antigens: PAA-**1** with different contents of attached ligand **1**. The screening of biotin-**1** conjugates loaded onto avidin-coated wells showed low signals. The bars represent the mean values with standard deviation (SD).

Commercially available diagnostic plates with loaded ASCA antigen and the plates coated with PAA and biotinylated conjugates of ligand **1** were examined via ELISA using the calibrant samples (2, 20, and 200 U/mol) and control sera (positive and negative) provided in the ASCA diagnostic kit (Figure 5). The optical density for ASCA-positive samples decreased with a decrease in the content of ligand **1** in PAA conjugates, while biotinylated ligand **1** did not differentiate between positive and negative serum samples. This observation may be due to the low amount of ligand **1** on the surface of the well, resulting in the inability to form detectable signals. On the contrary, the results obtained using synthetic PAA-based conjugates permitted the detection of much better signals, which depended on the degree of conjugation of carbohydrate ligand **1**. The conjugate with the highest loading of 20% demonstrated a slightly better result compared with commercially available plates coated with natural ASCA antigen.

**FIGURE 6:**
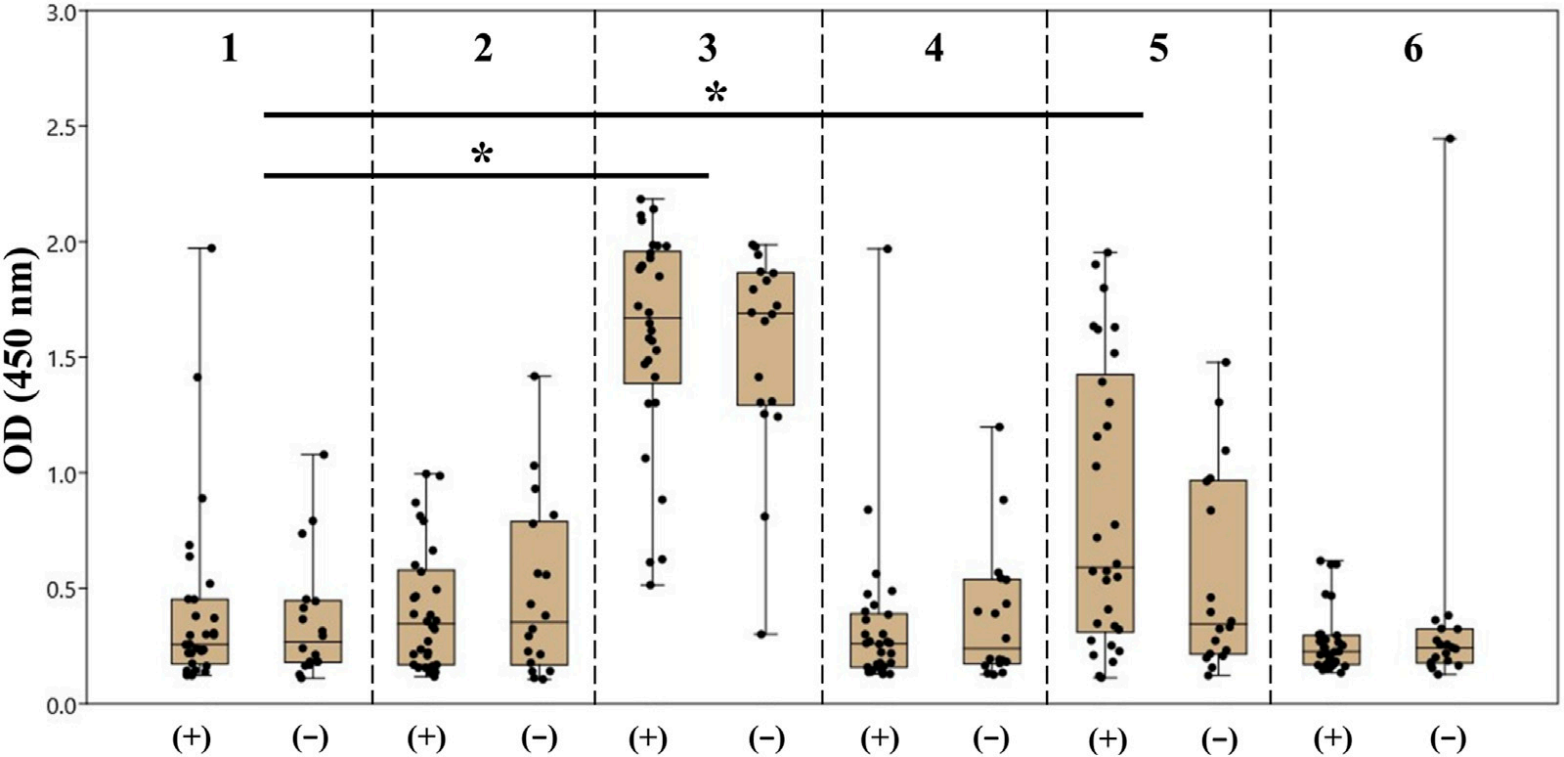
Screening results of the antibodies of the IgG class specific to oligosaccharides **1**–**6** in the blood sera of patients with colorectal cancer (+) and healthy donors (−). The data are presented as the box plot: the median value, upper and bottom quartiles, and upper and bottom boundaries are given for each antigen. The dots on the plot correspond to the average absorbance at 450 nm (OD450) in the ELISA for each serum sample. The sets of values were compared by the Mann–Whitney method (*p < 0.05, ns *p* > 0.05).

Taking into account the obtained results, we also prepared PAA conjugates of synthetic oligosaccharides **2–6** with 20% molar content of incorporated ligands and applied them to screen antibodies in the sera of healthy donors (N = 18) and patients with colorectal cancer (N = 30). The information about patients and healthy donors is summarized in Supplementary Tables S1, S2. The raw screening data are provided in Supplementary Table S3. The spread of the obtained results is presented as а box plot demonstrating median optical density and quartiles (Figure 6).

It should be noted that the formal replacement of the terminal α-(1→3)-mannoside residue with the β-(1→2)-mannoside one (**1** → **3**) or addition of the α-(1→2)-mannose residue to the non-reducing end of the original tetramannoside (**1** → **5**) significantly increased the dispersion and median value of observed optical density for both groups of samples. The result of comparison between carbohydrate ligands **1**–**6** with one-way ANOVA is provided in Supplementary Table S4. Of note, the median value and dispersion of optical density measured using ASCA tetramannoside **1** were the same for the serum samples of colorectal cancer patients and healthy donors. Alternatively, ligand **5** showed statistical relevance to an elevated median and wider dispersion for sera from patients with colorectal cancer (Table 1). It is noticeable that the observed level of anti-**5** IgG was higher in the sera of colorectal patients. The reason for the observed phenomenon should be further studied in the larger cohorts.

**TABLE 1:** Median values of antibody levels (OD) to synthetic oligosaccharides 1–6 for the two groups and the result of their comparison using the Mann–Whitney test.

Ligand	1		2		3		4		5		6	
Group	+	−	+	−	+	−	+	−	+	−	+	−
Median	0.26	0.27	0.35	0.35	1.67	1.69	0.35	0.35	0.59	0.34	0.23	0.24
*p*-value	0.96		0.82		0.51		0.83		0.091		0.87	

## 4 Discussion

The type of antigen coating and its immobilization protocol have a dramatic impact on ELISA results. Immobilization via biotin–avidin or biotin–streptavidin pairs is widely used and has certain advantages (Komarova et al., 2018; Matveev et al., 2018; Matveev et al., 2019; Kazakova et al., 2020; Krylov and Nifantiev, 2020; Wong et al., 2020). The streptavidin molecule is composed of four biotin-binding subunits, with an average distance of approximately 2.5 nm between biotin molecules. For a standard commercial microtiter plate with a biotin-binding capacity of 5 pmol per well, the calculated average distance between biotin-binding streptavidin subunits is 6.1 nm, assuming their uniform distribution (Krylov and Nifantiev, 2020). These distances exceed the size of the immobilized oligosaccharide molecules and exclude the interaction of neighboring ligands. Nevertheless, carbohydrate chains tightly attached to the polyacrylamide matrix are intertwined with each other, which may be attributed to their new antigenic properties. We found that PAA conjugates with 20% carbohydrate loading are more suitable for detecting ASCA-related antibodies.

We hypothesize that the high density of oligosaccharide ligands on the surface promotes the formation of multicenter interactions with several binding sites in immunoglobulins G and M. High-density carbohydrate screens have the advantage of the fixation of low-affinity antibodies. Moreover, low-affinity IgM antibodies with 10 binding centers are more easily detectable than IgG antibodies with two binding centers. Nevertheless, carbohydrate displays with low ligand density are more suitable for fine differentiation of antigenic properties of different ligands (Solovev et al., 2023).

The PAA conjugates of such type were used to form an array of oligosaccharides related to ASCA tetramannoside. Following the pioneering works by B. Sendid, D. Poulain, J. M. Mallet, and other studies based on the use of synthetic oligosaccharides, we investigated how the structural modifications in α-mannan chains affect their antigenicity. Particularly, we showed, for the first time, that the elongation of the ASCA epitope (α-Man-(1→3)-Man-α-(1→2)-Man-α-(1→2)-Man-α-) with an additional α-(1→2)-linked mannose residue significantly increases its recognition by human serum antibodies that can be associated with its higher immunogenicity. The high level of antibodies against oligomannoside 3 with β-(1→2)-linkage confirms the high immunogenicity of the corresponding epitope that is present in the polysaccharides of the natural fungal microbiota of the human intestine, which correlates with the previously reported results (Krylov et al., 2018; Sendid et al., 2021; Singh et al., 2023). The elevated median level of optical density for patients with colorectal cancer versus healthy donors was observed only for oligosaccharide **5** (Table 1). Of note, this ligand was recently introduced as a new high-affinity ligand for DC-SIGN, a basic receptor of the innate immune system (Krylov et al., 2023). Due to the low sensitivity and specificity of existing screening methods, much effort is focused on finding new potential biomarkers for colon cancer (Jimenez-Luna et al., 2021). Tumor and normal tissues are characterized by different glycosylation profiles (Zhao et al., 2018); in particular, high-mannose N-glycans are regarded as markers of malignant progression in the early stages of colorectal cancer (Boyaval et al., 2022). Considering these factors, deeper investigation of the immunological properties of mannosides is required, which may open new opportunities for improving existing ELISA test systems, as well as developing fungal diagnostic kits with new properties.

## Data Availability

The original contributions presented in the study are included in the article/Supplementary Material; further inquiries can be directed to the corresponding authors.
