# Supplementary material for: ASCA-related antibodies in the blood sera of healthy donors and patients with colorectal cancer: characterization with oligosaccharides related to Saccharomyces cerevisiae mannan
Source: Front Mol Biosci. 2023 Dec 7;10:1296828. doi: 10.3389/fmolb.2023.1296828 (PMC10749338; doi:10.3389/fmolb.2023.1296828)
Supplement: Supplementary file 1 [file Table1.DOCX]

Supplementary Material

# Supplementary Tables

**Table S1.** Patient characteristics.

| **Patients with CRC** | **Sex** | **Age** | **Location** | **Histological types** | **TNM** | **Stage** |
| --- | --- | --- | --- | --- | --- | --- |
| Patient 1 | M | 54 | Sigmoid colon | adenocarcinoma | T2N0M0 | I |
| Patient 2 | M | 69 | Sigmoid colon | metastatic adenocarcinoma | T1N1M0 | III |
| Patient 3 | F | 73 | Straight intestine | adenocarcinoma | T3N1bM0 | IIIb |
| Patient 4 | F | 59 | Straight intestine | mucinous adenocarcinoma | T4aN0M1 | IV |
| Patient 5 | F | 63 | Sigmoid colon | adenocarcinoma | T3N2аM0 | III |
| Patient 6 | M | 43 | Straight intestine | adenocarcinoma | T3N1aM0 | IIIa |
| Patient 7 | F | 37 | Blind intestine | adenocarcinoma | T3N1M1 | IV |
| Patient 8 | F | 68 | Straight intestine | adenocarcinoma | T4аN1аM0 | IIIb |
| Patient 9 | F | 33 | Sigmoid colon | adenocarcinoma | Т3N1bM0 | IIIb |
| Patient 10 | F | 63 | Blind intestine | adenocarcinoma | T3N0M0 | IIa |
| Patient 11 | M | 59 | Ascending part of the colon | adenocarcinoma | T3N0M0 | IIa |
| Patient 12 | F | 69 | Ascending part of the colon | adenocarcinoma | Т3N0M0 | II |
| Patient 13 | M | 69 | Ascending part of the colon | adenocarcinoma | Т3N0M0 | II |
| Patient 14 | M | 64 | Sigmoid colon | adenocarcinoma | T1N0M0 | I |
| Patient 15 | F | 72 | Discending part of the colon | adenocarcinoma | T3N1bM0 | IIIb |
| Patient 16 | F | 54 | Ascending part of the colon | adenocarcinoma | T3N0M1a | IV |
| Patient 17 | F | 60 | Straight intestine | adenocarcinoma | T3N1M0 | IIa |
| Patient 18 | M | 65 | Discending part of the colon | adenocarcinoma | T3N1bM0 | IIIb |
| Patient 19 | M | 63 | Sigmoid colon | adenocarcinoma | T3N1M0 | III |
| Patient 20 | M | 67 | Sigmoid colon | adenocarcinoma | T4bN1M1b | IVb |
| Patient 21 | F | 67 | Discending part of the colon | adenocarcinoma | T4аN1M0 | IIIb |
| Patient 22 | M | 56 | Discending part of the colon | adenocarcinoma | T2N0M0 | I |
| Patient 23 | F | 71 | Ascending part of the colon | adenocarcinoma | T3N0M0 | IIa |
| Patient 24 | M | 64 | Sigmoid colon | adenocarcinoma | T3N1bM0 | IIIb |
| Patient 25 | M | 58 | Discending part of the colon | adenocarcinoma | T3N1M1 | IVa |
| Patient 26 | F | 65 | Sigmoid colon | adenocarcinoma | Т3N0M0 | IIa |
| Patient 27 | M | 39 | Blind intestine | adenocarcinoma | T3N1сM1 | IVc |
| Patient 28 | M | 58 | Sigmoid colon | adenocarcinoma | T3N0M0 | IIa |
| Patient 29 | M | 52 | Sigmoid colon | adenocarcinoma | T3N0M0 | IIa |
| Patient 30 | F | 39 | Blind intestine | adenocarcinoma | T3N1M0 | III |

**Table S2.** Control group characteristics.

| **Control** | **Sex** | **Age** |
| --- | --- | --- |
| Control 1 | M | 66 |
| Control 2 | F | 50 |
| Control 3 | M | 52 |
| Control 4 | F | 61 |
| Control 5 | M | 41 |
| Control 6 | F | 54 |
| Control 7 | F | 70 |
| Control 8 | F | 60 |
| Control 9 | M | 71 |
| Control 10 | F | 48 |
| Control 11 | M | 28 |
| Control 12 | M | 40 |
| Control 13 | F | 65 |
| Control 14 | F | 23 |
| Control 15 | M | 19 |
| Control 16 | M | 37 |
| Control 17 | M | 33 |
| Control 18 | F | 20 |

**Table S3**. Raw-data of sera screening with ligands **1-6**.

| **Ligand** | | **1** | **2** | **3** | **4** | **5** | **6** |
| --- | --- | --- | --- | --- | --- | --- | --- |
| **Patient** | **1** | 0.6865 | 0.3573 | 1.2999 | 0.1627 | 1.6299 | 0.6039 |
|  | **2** | 0.3711 | 0.3846 | 2.1413 | 0.2583 | 1.9013 | 0.3010 |
|  | **3** | 0.2560 | 0.7916 | 1.3030 | 0.3005 | 0.7751 | 0.1569 |
|  | **4** | 0.1747 | 0.1631 | 0.6125 | 0.1560 | 0.3362 | 0.6031 |
|  | **5** | 0.6380 | 0.1688 | 1.8965 | 0.3996 | 1.7992 | 0.2779 |
|  | **6** | 0.2397 | 0.4942 | 1.9818 | 0.5628 | 0.5741 | 0.4675 |
|  | **7** | 0.2340 | 0.1373 | 1.9288 | 0.2698 | 0.7194 | 0.2248 |
|  | **8** | 0.2193 | 0.4587 | 1.8496 | 0.1411 | 1.6344 | 0.4741 |
|  | **9** | 0.3003 | 0.2094 | 1.7213 | 0.4745 | 0.5747 | 0.2270 |
|  | **10** | 0.1234 | 0.1557 | 0.6251 | 0.1394 | 0.1206 | 0.2148 |
|  | **11** | 0.3799 | 0.1364 | 1.6927 | 0.4883 | 0.2742 | 0.2707 |
|  | **12** | 0.1434 | 0.2358 | 0.5141 | 0.1289 | 0.1816 | 0.1626 |
|  | **13** | 0.4533 | 0.6639 | 2.1839 | 0.2622 | 1.6202 | 0.2995 |
|  | **14** | 0.1637 | 0.1694 | 0.8834 | 0.1457 | 0.1125 | 0.1676 |
|  | **15** | 1.4132 | 0.8704 | 1.8808 | 0.2629 | 1.5174 | 0.2128 |
|  | **16** | 0.1374 | 0.3362 | 1.4701 | 0.1588 | 0.4088 | 0.1467 |
|  | **17** | 0.3068 | 0.3595 | 1.8898 | 0.4276 | 1.0279 | 0.2114 |
|  | **18** | 0.1275 | 0.2227 | 1.5300 | 0.1292 | 0.2107 | 0.1704 |
|  | **19** | 0.2204 | 0.6005 | 1.0626 | 0.2683 | 0.6059 | 0.2438 |
|  | **20** | 0.4522 | 0.3886 | 1.4866 | 0.1767 | 1.2015 | 0.2957 |
|  | **21** | 0.1446 | 0.1564 | 1.6154 | 0.1370 | 0.3213 | 0.2425 |
|  | **22** | 0.1229 | 0.1176 | 1.5815 | 0.2184 | 0.3476 | 0.1503 |
|  | **23** | 0.8895 | 0.2144 | 1.9501 | 0.8397 | 1.1565 | 0.2528 |
|  | **24** | 1.9720 | 0.9953 | 2.1142 | 1.9683 | 1.9532 | 0.6191 |
|  | **25** | 0.5200 | 0.5717 | 2.0912 | 0.2226 | 1.3934 | 0.2667 |
|  | **26** | 0.2983 | 0.2706 | 1.9855 | 0.3016 | 1.3042 | 0.2029 |
|  | **27** | 0.2340 | 0.3230 | 1.9805 | 0.3863 | 0.5350 | 0.1820 |
|  | **28** | 0.2576 | 0.4654 | 1.6472 | 0.3637 | 0.2520 | 0.1684 |
|  | **29** | 0.2969 | 0.9866 | 1.5708 | 0.1776 | 0.5488 | 0.1834 |
|  | **30** | 0.2283 | 0.8128 | 1.4142 | 0.1708 | 0.2277 | 0.1341 |
| **Control** | **1** | 0.1652 | 0.2932 | 0.3007 | 0.1318 | 0.1569 | 0.2192 |
|  | **2** | 0.1821 | 1.4176 | 1.6566 | 0.1920 | 0.2165 | 0.1670 |
|  | **3** | 0.4148 | 0.2139 | 1.9434 | 0.4328 | 0.4603 | 0.2015 |
|  | **4** | 0.2941 | 0.1414 | 1.8638 | 0.5677 | 0.3243 | 0.1654 |
|  | **5** | 0.2407 | 0.1064 | 0.8104 | 0.1882 | 0.1986 | 0.1540 |
|  | **6** | 0.2126 | 0.5582 | 1.3041 | 0.1653 | 0.3570 | 0.1796 |
|  | **7** | 0.1265 | 0.8170 | 1.4142 | 0.1265 | 0.3327 | 0.1864 |
|  | **8** | 0.3148 | 0.7796 | 1.8705 | 0.4006 | 1.0960 | 0.3824 |
|  | **9** | 0.7365 | 0.9308 | 1.9776 | 1.1979 | 0.9758 | 0.2381 |
|  | **10** | 0.1111 | 0.1117 | 1.7231 | 0.1352 | 0.1225 | 0.1269 |
|  | **11** | 0.1693 | 0.1402 | 1.3082 | 0.1821 | 0.2734 | 0.2564 |
|  | **12** | 1.0782 | 1.0307 | 1.8318 | 0.5444 | 1.4782 | 2.4460 |
|  | **13** | 0.4445 | 0.5642 | 1.2544 | 0.1759 | 0.3972 | 0.3238 |
|  | **14** | 0.3662 | 0.3240 | 1.2422 | 0.1942 | 1.3049 | 0.3623 |
|  | **15** | 0.4523 | 0.4313 | 1.7934 | 0.5371 | 0.8365 | 0.2744 |
|  | **16** | 0.7920 | 0.2266 | 1.6861 | 0.8826 | 0.2317 | 0.3251 |
|  | **17** | 0.1824 | 0.3819 | 1.6935 | 0.3911 | 0.2075 | 0.2440 |
|  | **18** | 0.1829 | 0.1771 | 1.9869 | 0.2837 | 0.9629 | 0.2565 |

**Table S4**. One-way ANOVA test p-values for six carbohydrate ligands **1-6**

| **Ligand** | **1** | **2** | **3** | **4** | **5** | **6** |
| --- | --- | --- | --- | --- | --- | --- |
| **1** | 1 | 0.46516 | <0.00001 | 0.62272 | 0.000449 | 0.26157 |
| **2** | 0.46516 | 1 | <0.00001 | 0.19765 | 0.001724 | 0.053081 |
| **3** | <0.00001 | <0.00001 | 1 | <0.00001 | <0.00001 | <0.00001 |
| **4** | 0.622715 | 0.19765 | <0.00001 | 1 | 0.000091 | 0.50211 |
| **5** | 0.000449 | 0.001724 | <0.00001 | 0.000091 | 1 | 0.000017 |
| **6** | 0.261572 | 0.053081 | <0.00001 | 0.50211 | 0.000017 | 1 |
